# Identifying malaria elimination strategies in the presence of human movement in Bangladesh

**DOI:** 10.1038/s43856-025-01145-6

**Published:** 2025-11-07

**Authors:** Ayesha S. Mahmud, Meng-Chun Chang, Daniel T. Citron, Kenth Engø-Monsen, Abdullah Abu Sayeed, Sazid Ibna Zaman, Didar Uddin, Md Mushfiqur Rahman, Mosiqure Rahaman, Md Nazmul Islam, Richard James Maude, Caroline O. Buckee, Hsiao-Han Chang

**Affiliations:** 1 Department of Demography, University of California Berkeley, Berkeley, CA USA; 2 Institute of Bioinformatics and Structural Biology and Department of Life Science, National Tsing Hua University, Hsinchu, Taiwan; 3 Department of Population Health, NYU Grossman School of Medicine, New York, NY USA; 4 Smart Innovation Norway AS, Halden, Norway; 5 Chittagong Medical College and Hospital, Chattogram, Bangladesh; 6 Mahidol-Oxford Tropical Medicine Research Unit, Faculty of Tropical Medicine, Mahidol University, Bangkok, Thailand; 7 National Malaria Elimination Program and ATDs Control Program, Communicable Disease Control, Directorate General of Health Services, Dhaka, Bangladesh; 8 Communicable Disease Control, Directorate General of Health Services, Dhaka, Bangladesh; 9 Centre for Tropical Medicine and Global Health, Nuffield Dept of Medicine, University of Oxford, Oxford, UK; 10 Open University, Milton Keynes, UK; 11 The Center for Communicable Disease Dynamics and Department of Epidemiology, Harvard T.H. Chan School of Public Health, Boston, MA USA

**Keywords:** Malaria, Disease prevention

## Abstract

**Background:**

Malaria transmission in the Chittagong Hill Tracts (CHT) districts in Bangladesh is characterized by considerable heterogeneity in incidence and the frequent mixing and importation of parasites across districts. Thus, elimination efforts must account for human mobility between endemic and non-endemic locations, and the relative importance of local transmission and parasite importation domestically.

**Methods:**

We construct a metapopulation malaria model, parameterized by human mobility data and fit to epidemiological data, to guide elimination efforts in the region.

**Results:**

We find substantial heterogeneity in the transmission intensity across the CHT, with the estimated basic reproduction number varying greatly across places with similar levels of observed incidence. When vector control interventions are applied locally, the greatest impact in reducing overall incidence are in places with both high transmission intensity and high connectivity with more populated districts in the western part of the CHT.

**Conclusions:**

Local elimination in several areas with low or intermediate incidence has a moderate impact in reducing overall incidence, indicating that only focusing on high incidence areas is not sufficient for malaria elimination. More generally, our modeling framework can be used to prioritize resource allocation and identify the conditions necessary for malaria elimination in the region.

## Plain Language Summary

Malaria can be hard to eliminate because people move between regions. This movement can carry parasites from areas with high transmission (“source” regions) into areas with lower transmission (“sink” regions). We built a mathematical model of malaria in Bangladesh that combines human movement patterns with disease data. We found that elimination works best when control efforts target areas that have both high malaria transmission and strong connections to larger, more populated districts. Focusing only on areas with high numbers of cases is not enough to achieve elimination. Our model can facilitate public health decisions on where to focus resources and what conditions are needed to achieve malaria elimination in Bangladesh.

## Abstract (web summary)

Mahmud, Chang et al., model malaria transmission encompassing human mobility and epidemiological data in Bangladesh to guide elimination efforts. Local vector control efforts have the greatest impact when targeted to places with both high transmission intensity and high connectivity.

## Introduction

While the number of malaria cases in Bangladesh has decreased dramatically from 2008 to 2020, the Chittagong Hill Tracts (CHT) region in the southeast of the country still bears a substantial burden of the disease^1–3^. Human mobility complicates control and elimination efforts for malaria by facilitating the spread of the parasite between regions with different levels of transmission and incidence. Frequent travel between endemic and non-endemic regions, along with the presence of asymptomatic carriers of malaria, make it challenging to fully prevent the reintroduction and re-establishment of malaria parasites into regions with no local infections^4^. In Bangladesh, a study based on a travel survey of 2090 malaria patients revealed substantial human mobility within the CHT region^5^. Travel patterns included both short-term daily trips and longer stays, with substantial variation based on purpose, demographics, and occupation. Reported travel between coastal, more densely populated regions and forested areas with higher number of malaria cases likely plays an important role in malaria transmission.

To achieve the goal of elimination it can help to identify regions that are “sources”, i.e., where humans travel to and become infected, and “sinks”, i.e., where the parasite is introduced by travelers, since the most effective intervention methods for source and sink populations may differ. Common malaria interventions include vector control strategies such as insecticide-treated nets (ITNs) and indoor residual spraying (IRS), as well as treatment strategies like rapid diagnostic testing and treatment, and intermittent preventive treatment (IPT) for vulnerable populations^6^. Testing and treating of travelers moving to and from endemic source areas, or implementing vector control efforts in these areas, has the potential to dramatically reduce transmission and incidence across the CHT. Thus, to allocate resources appropriately for such a strategy, we need to identify source and sink regions based on the local malaria transmission intensity and the connectivity between locations. This is challenging since patients may not be diagnosed in the place of infection and using local incidence rates to identify source and sink locations can potentially be misleading.

Malaria incidence in the CHT is highly heterogeneous, with the highest incidence occurring along the forested border regions in the southeast^1^. However, in a region with a highly mobile population, the geographic heterogeneity in incidence rates may not reflect underlying patterns of transmission. Previous work has shown that while the forested regions were contributing imported infections to the lower transmission areas in the southwest, there was substantial importation of parasites throughout the CHT^7^. These results suggest that human mobility and mixing across the CHT continue to pose a major challenge to elimination, and that the efficacy of intervention efforts is dependent on both the levels of parasite importation and local transmission in a location. Due to these complexities, mapping parasite sources and quantifying local transmission intensity can be greatly facilitated by a mechanistic modeling framework that incorporates human mobility and transmission dynamics within and across locations. Here we construct a metapopulation human-mosquito malaria model, parameterized by human mobility data and fit to epidemiological data^8,9^, to quantify the local basic reproduction number, R_0_, and the impact of targeted vector control interventions.

Mobile phone call detail records (CDR) offer a unique opportunity to measure human mobility at spatial and temporal scales relevant for disease transmission and has been used previously to map sources and sinks of malaria parasites^8,10,11^. Yet the sparsity of mobile phone towers in low density population in forest and forest fringe areas, where vector density tends to be high^12^, means that measuring movement to and from these regions remains difficult. To address this issue, we first used a gravity model, fitted to mobile phone data from areas with high coverage, to infer mobility in regions without sufficient coverage of phone towers. The complete mobility data was then used to parameterize a mechanistic metapopulation model for the spatial transmission of malaria in the CHT. We find that malaria transmission intensity varies greatly across location in the CHT. The fitted model was used to identify the most important locations for vector control efforts. Our results show that the greatest impact in reducing overall incidence through local interventions, are in places with both high transmission intensity and high connectivity with more populated districts in the western part of the CHT. Overall, our methods provide a framework for targeted public health resource allocation in regions with heterogeneous transmission and high connectivity.

## Methods

### Malaria data

The malaria incidence data (number of recorded cases per 1000 persons per year) were collected by the National Malaria Elimination Programme from January 2015 to August 2018^7^ and the population sizes were from the census in 2011^13^.

### Human movements

We estimated human movements between all pairs of unions (smallest administrative unit in Bangladesh) with at least one mobile phone tower using mobile phone call data records from April 1st to September 30th, 2017^7^. Subscribers were assigned locations based on the union where their most frequently contacted mobile phone tower was located on a given day. Movements were identified if the primary location changed from 1 day to the next. We first calculated the average numbers of daily movements among unions, then obtained the proportion of trips from union *i* to union *j*, denoted as *p*_*ij*_, by dividing the average number of daily trips from union *i* to *j* by the total number of daily trips originating from union *i*. Since human movements to and from unions without any mobile phone towers could not be estimated from the mobile phone calling data—these unions are primarily located in forested areas with higher malaria incidence rates—we trained a statistical model based on estimated human movements from the mobile calling data to estimate human movements for unions without any mobile phone towers. We used a modified version of the gravity model, fit to observed data for locations with mobile phone towers, to estimate the movement between unions that were missing towers, *M*_ij_. Specifically, we assumed *M*_ij_ ~ Poisson(*λ*_ij_) and fit the following Poisson regression model:

1 $$log ({\lambda }_{{{{\rm{ij}}}}})={\alpha }_{{{{\rm{i}}}}}+{\alpha }_{{{{\rm{j}}}}}+{\beta }_{1}{Log}({H}_{{{{\rm{i}}}}})+{\beta }_{2}{Log}({H}_{{{{\rm{j}}}}})+{\beta }_{3}{T_{ij}}$$

where *λ*_ij_ is the expected number of trips from union *i* to union *j*; *α*_i_ and *α*_j_ are fixed-effects at the district-level for unions *i* and *j*, respectively; *H*_*i*_ and *H*_*j*_ are the population sizes of unions *i* and union *j*, respectively; and *T*_*ij*_ is the travel time between union *i* and union *j*^14^. We used the *glm* package in *R* to estimate the parameters, and used the fitted model to estimate the unobserved *M*_ij_.

We also estimated the number of subscribers who remain in the same union, i.e. the number who do not move in a given day, based on a similar model fit to observed data for locations with mobile phone towers. We added income as a covariate to the model and fit the following Poisson regression model:

2 $$log ({\lambda }_{{{{\rm{ii}}}}})=\alpha +{\beta }_{1}{Log}({H}_{{{{\rm{i}}}}})+{\beta }_{2}{{Inc}}_{{{{\rm{i}}}}}$$

where *λ*_ii_ is the expected number of subscribers who remain in a given union; *α* is the fixed-effect at the district-level for union *i*; *H*_*i*_ is the population size of union *i*, as defined in Eq. 1; and *Inc*_*i*_ is the average income of union *i* from^15^. Missing *p*_*ij*_ values were then calculated according to $\frac{{\lambda }_{{ij}}}{{\sum }_{j}{\lambda }_{{ij}}}.$

### Metapopulation malaria transmission model

We used a metapopulation malaria transmission model, developed by Ruktanonchai et al.^8^, parameterized by the human mobility data described above. This model is an extension of the Ross-Macdonald framework, which has been adapted to include human movement dynamics^8,16,17^. Within this model, we track the proportions of infected humans (*X*_*i*_) and mosquitoes (*Y*_*i*_) for each union *i*.

The model incorporates two main aspects of human mobility that affect malaria transmission. Firstly, it considers the impact of infected human travelers on the infection of susceptible mosquitoes in their destination. This is quantified by the variable ${\kappa }_{i}$, which represents the proportion of infected humans in location *i*, including both residents and visitors. Secondly, the model accounts for how infections of residents in each location *i* are influenced by exposure to infectious mosquitoes both locally and in locations they visit. This influence is quantified by aggregating the impacts from all locations, with each contribution weighted by the mobility estimates *p*_*ij*_.

3 $${\kappa }_{i}=\frac{{\sum }_{j}{p}_{{ji}}{X}_{j}{H}_{j}}{{\sum }_{j}{p}_{{ji}}{H}_{j}}$$

4 $$\frac{d{X}_{i}}{{dt}}={\sum }_{j}{p}_{{ij}}{m}_{j}{ab}{Y}_{j}\left(1-{X}_{i}\right)-{{rX}}_{i}$$

5 $$\frac{d{Y}_{i}}{{dt}}={ac}{\kappa }_{i}\left({e}^{-\mu \tau }-{Y}_{i}\right)-\mu {Y}_{i}$$

Here, *m* represents the ratio of the total female mosquito population to the total human population, *r* describes the rate at which infected humans recover, *μ* indicates the mortality rate of infected mosquitoes, and *τ* refers to the incubation period of the disease within mosquitoes. The biting rate of mosquitoes on humans is denoted by *a*, whereas *b* and *c* represent the probabilities that a bite from an infectious mosquito will successfully transmit the disease to a susceptible human and vice versa, respectively. *H*_*i*_ denotes the human population size in union *i*^13^. Among these parameters, *m* is more likely to be study-site specific, while other parameters representing features of the mosquito biology and malaria infection and transmission and are commonly assumed to be constant in previous studies^8^. Therefore, we used parameter values for *a*, *b*, *c*, *r*, *μ*, and *τ* from other malaria studies (see Supplementary Table 1) and solved for *m* for each union using the following approach.

Since mosquito dynamics are relatively faster compared to human dynamics, we assumed a quasi-equilibrium for infectious mosquitoes. We solved for quasi-equilibrium *Y*_*i*_ by setting Eq. 5 to zero, and substituted this value into Eq. 4 to derive the resulting equation for the proportion of infectious humans (Eq. 6) (see details in ref. ^8^). The solution for quasi-equilibrium *Y*_*i*_, *Y*_i_*, is $\frac{{ac}{\kappa }_{i}}{\mu +{ac}{\kappa }_{i}}{e}^{-\mu \tau }$.

6 $$\frac{d{X}_{i}}{{dt}}={\sum }_{j}{p}_{{ij}}{m}_{j}{ab}{Y}_{j}^{* }\left(1-{X}_{i}\right)-{{rX}}_{i}$$

Equation 6 describes how the proportions of infected humans change over time. We followed the methods developed by Ruktanonchai et al.^8^, and described briefly below, to estimate the vectoral capacity, *m*_*i*_, for each location. To solve for *m*_*i*_, we assume steady-state and set Eq. 6 to zero.

${\sum }_{j}{p}_{{ij}}{m}_{j}{ab}{Y}_{j}^{* }\left(1-{X}_{i}\right)-{{rX}}_{i}=0{\to }^{{\mbox{yields}}}{\sum }_{j}{p}_{{ij}}{m}_{j}{ab}{Y}_{j}^{* }=\frac{{{rX}}_{i}}{1-{X}_{i}}$, which can be expressed in matrix form as

7 $${AO}=g(X)$$

where

$$A=P{{{\rm{diag}}}}(\, f\left(X\right))$$

$$O=\left(\begin{array}{c}{O}_{1}\\ \vdots \\ {O}_{n}\end{array}\right)\; {{{\rm{with}}}}\; {O}_{i}=\frac{{m}_{i}{a}^{2}{e}^{-\mu \tau }}{\tau },$$

$$g\left(X\right)=\left(\begin{array}{c}{g}_{1}\left(X\right)\\ \vdots \\ {g}_{n}\left(X\right)\end{array}\right)\; {{{\rm{with}}}}\; {g}_{i}\left(X\right)=\frac{r{X}_{i}}{1-{X}_{i}}\; {{{\rm{and}}}}$$

$$f\left(X\right)=\left(\begin{array}{c}{f}_{1}\left(X\right)\\ \vdots \\ {f}_{n}\left(X\right)\end{array}\right) \; {{{\rm{with}}}}\; {f}_{i}\left(X\right)=\frac{{bc}{\kappa }_{i}\mu }{{ac}{\kappa }_{i}+\mu }.$$

Then *O* can be solved by

8 $$O={A}^{-1}g(X)$$

and

$${m}_{i}=\frac{{O}_{i}\tau }{{a}^{2}{e}^{-\mu \tau }}.$$

Since our observed data is malaria incidence by location, *I*_*i*_, we used the steady-state relationship between incidence and prevalence and set ${X}_{i}^{* }=\frac{{I}_{i}}{r}$. Thus, given the malaria incidence of each union, we calculated analytical solutions for *m*_*i*_ by solving for *O* (Eq. 8).

This analytical steady-state solution does not restrict values of *m*_*i*_ to be >zero. For regions with very low or zero incidence, it is possible for the solution of *m*_*i*_ to be negative. However, as negative *m*_*i*_ has no biological meaning, for the unions with derived *m*_*i*_ < 0, we replaced them by a small positive value (10^−10^). To ensure this did not influence the fitting to the incidence data, we compared the incidence predicted from this mechanistic model and the empirical incidence data, and found they were consistent (Supplementary Fig. 1).

For comparison, we also constructed the basic Ross-Macdonald model^16^ without spatial component as follows:

9 $$\frac{d{X}_{i}}{{dt}}={m}_{i}{ab}{Y}_{i}\left(1-{X}_{i}\right)-{{rX}}_{i}$$

10 $$\frac{d{Y}_{i}}{{dt}}={ac}{X}_{i}\left({e}^{-\mu \tau }-{Y}_{i}\right)-\mu {Y}_{i}$$

Similarly, we assumed quasi-equilibrium for *Y*_*i*_ by setting Eq. 10 to zero, substituting this value into Eq. 9, and then setting the modified Eq. 9 to zero to derive the expression for *m*_*nomob,i*_ without considering spatial dynamics:

11 $${m}_{{{{\rm{nomob}}}},\,i}=\frac{r{X}_{i}({ac}{X}_{i}+\mu )}{{a}^{2}{bc}{e}^{-\mu \tau }{X}_{i}(1-{X}_{i})}$$

With two versions of *m*_*i*_, one considering mobility and one without, we derived two corresponding versions of *R*_0_ for each union *i* by

12 $${R}_{0i}=\frac{{m}_{i}{a}^{2}{bc}{e}^{-\mu \tau }}{r\mu }.$$

In Eq. 6, the changes in the proportion of infectious humans in location *i* are driven by infectious mosquitoes either in location *i* (${p}_{{ii}}{m}_{i}{ab}{Y}_{j}^{* }$) or in location *j* (${p}_{{ij}}{m}_{j}{ab}{Y}_{j}^{* }$). To compute the proportion of infected individuals whose residential location is union *i* and who were infected either while staying in union *i* (*C*_*ii*_) or while traveling to union *j* (*C*_*ij*_), each term is divided by the sum of all terms as follows:

13 $${C}_{{ij}}=\frac{{p}_{{ij}}{m}_{j}{ab}{Y}_{j}^{* }}{{\sum }_{k=1}^{n}{P}_{{ik}}{m}_{k}{ab}{Y}_{k}^{* }}$$

The number of unions is denoted by *n*. The proportion of imported infections (can be viewed as “sink score”) in union *i* is then calculated by ${\sum }_{j=1\,(j\ne i)}^{n}{C}_{{ij}}$. The “source score” for union *i* is equal to ${\sum }_{j=1\,(j\ne i)}^{n}{H}_{j}{I}_{j}{C}_{{ji}}$, where *I*_*j*_ is the incidence rate in location *j*.

Finally, we defined highly populated areas (*H*_high_) as those with population sizes in the top quartile. We quantified the connectivity to these areas for each union by summing the number of people traveling to or from the highly populated areas as follows:

14 $${D}_{i}={H}_{i}{\sum }_{j\in {H}_{{{\mbox{high}}}}}{P}_{{ij}}+{\sum }_{j\in {H}_{{{\mbox{high}}}}}^{n}{H}_{j}{P}_{{ji}}$$

To identify malaria elimination strategies, we simulated the impact of local elimination on overall reduction of incidence in the region. We did so by setting the mosquito-to-human ratio for each union to zero one at a time, which is analogous to the maximum level of mosquito control. The effect of interventions was calculated by the reduction in the number of infected individuals across the whole CHT region.

### Ethical approval

Ethical approval was obtained from the Oxford Tropical Research Ethical Committee (1-15), Bangladesh Medical Research Council Ethical Committee (BMRC/NREC/2013-2016/1154) and Harvard University Human Research Protection Program (IRB14-2669). No consent was required as we used anonymized routinely collected malaria surveillance data which was aggregated as numbers of cases with no personally identifiable information. Ethical approval to use this dataset was approved by all three committees.

### Reporting summary

Further information on research design is available in the Nature Portfolio Reporting Summary linked to this article.

## Results

**Fig. 1:**
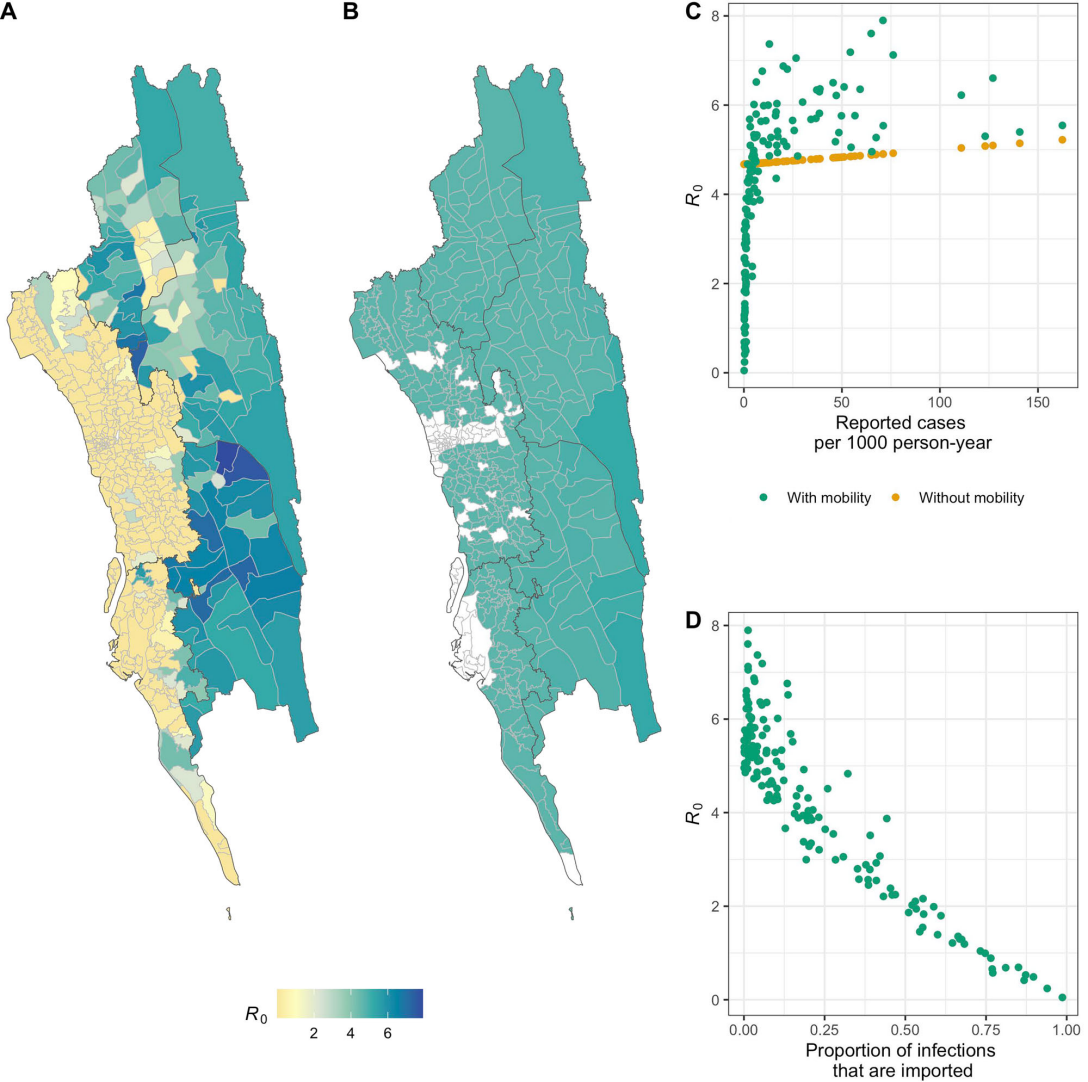
Variation in local transmission intensity. Estimated local transmission intensity (R_0_) estimated (**A**) with and (**B**) without mobility data included in the mechanistic transmission model (i.e. with and without spatial coupling between locations). **C** Estimated local transmission versus incidence (number of recorded cases per 1000 persons per year). R_0_ estimated without spatial coupling between locations is proportional to the local observed incidence; R_0_ estimated with spatial coupling can vary widely across locations with similar levels of incidence. **D** Relative importance of local transmission versus proportion of infections that are imported for each location.

We adapted a metapopulation mechanistic model of malaria transmission^8^ to quantify parasite importation and level of local transmission of malaria in the CHT in Bangladesh. Spatial coupling between locations was informed by human mobility data derived from mobile phone CDR. For regions without sufficient mobile phone coverage, we used an adapted gravity model, where travel volume between pairs of locations is estimated as a function of the size of the locations and the distance between them (see Materials and Methods), to infer the missing mobility data (Supplementary Fig. 2). We estimated the transmission intensity for each union—as quantified by the local reproduction number, R_0_,—by fitting the mechanistic model steady-state simulations to observed incidence data (Supplementary Fig. 3). We compared the results of using a metapopulation model with spatial coupling between unions with a classical model with no spatial coupling, and much greater heterogeneity in transmission was found in the CHT when human mobility was included in the model (Fig. 1A). When spatial coupling between locations was ignored, the estimated transmission intensity was directly proportional to the incidence; after considering mobility, transmission intensity was correlated but not always proportional to the incidence (Fig. 1B and Supplementary Fig. 3). While incidence was highest in the forested southwestern region, we found that transmission intensity in that area was highly heterogeneous; locations with the highest incidence were not necessarily the same areas with the highest local transmission intensity (Fig. 1). Our results also showed that pockets of high transmission intensity exist in the northern part of the CHTs, which is an area with relatively low incidence.

**Fig. 2:**
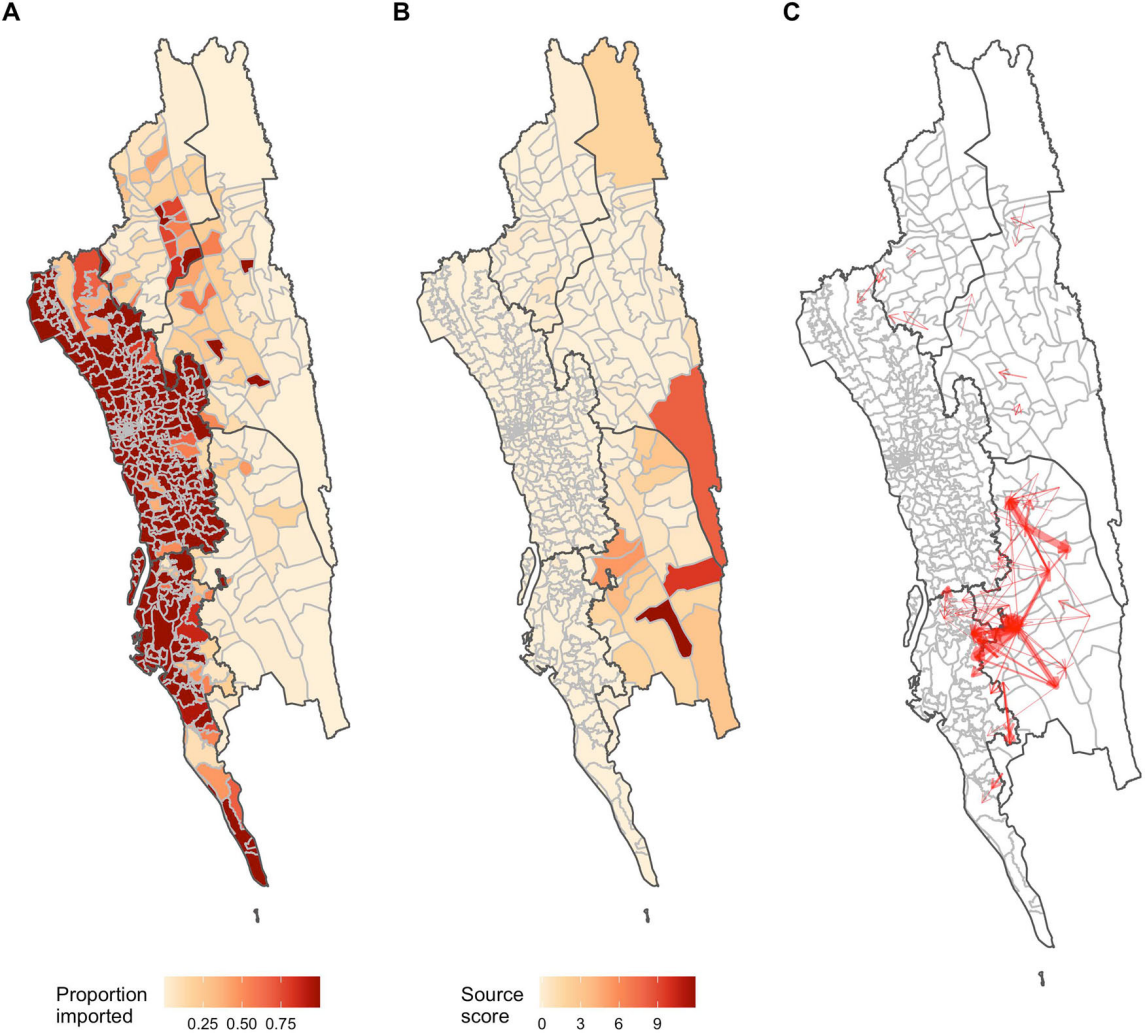
Source and sink dynamics in the CHT. **A** The estimated proportion of imported infections; (**B**) the source score; and (**C**) the top 0.05% of transmission routes (the width of the arrows is proportional to the volume of importation).

A key advantage of using a mechanistic metapopulation model of transmission is that it allows us to quantify the relative importance of local transmission relative to imported infections, a crucial component of guiding elimination efforts. Using the mechanistic model with complete mobility data covering all unions in the CHT, we calculated the source and sink scores for each location and identified the top transmission routes (Fig. 2). The source score quantifies the contribution of each location to infections that occur elsewhere; the sink score is the proportion of imported infections in each location. We found that source populations were mainly located in the southeastern part of the CHT (Fig. 2B); these regions had relatively high incidence and were well-connected to more densely populated unions in the western part of the CHT. Nine out of the top ten source locations were in the heavily forested Bandarban district in the southeast. Unions in the western part of the CHT had lower local transmission intensity and a higher proportion of infections that were imported (Fig. 2).

**Fig. 3:**
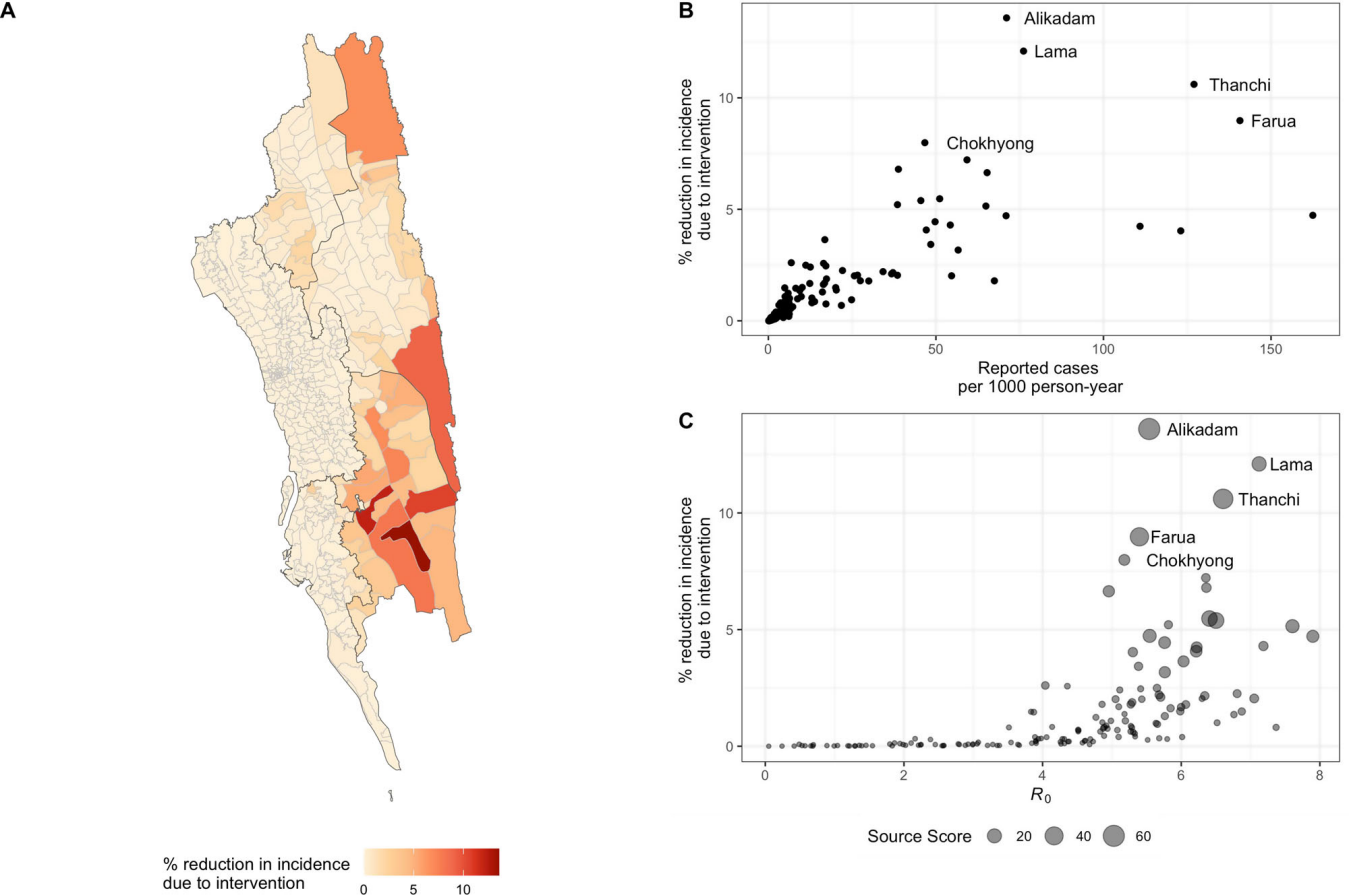
Impact of local elimination. **A** Map showing the impact of local intervention in reducing overall incidence in the CHT; (**B**) the impact of the intervention by incidence (number of recorded cases per 1000 persons per year) of the location where the intervention is administered; and by (**C**) the local transmission intensity and source score. The top five unions where the intervention led to highest overall reduction in incidence are labeled in (**B**, **C**).

We quantified the effect of public health measures by applying vector-control interventions, resulting in local elimination of the mosquito population, to one union at a time, with the goal of understanding how to prioritize limited public health resources. While the effect of the intervention—measured as the percentage reduction in the overall incidence across the entire region—was positively correlated with the estimated transmission intensity (correlation = 0.541; *p* < 10^-12^) and incidence (correlation = 0.796, *p* < 2.2 × 10^-16^) of the union where the intervention was applied, the effect size varied considerably for locations with similar R_0_ and incidence (Fig. 3). This is due to the impact of human movements and malaria source-sink dynamics between locations. The unions that had the highest impact, such as Alikadam and Lama, were those with high source scores as well as relatively high local transmission and incidence (Fig. 3). In general, the effect of the intervention was higher when implemented in unions with higher source scores, higher connectivity to highly populated areas, and lower proportion of imported infections (Spearman’s partial correlation = 0.91 [source], 0.46 [connectivity], and -0.37 [prop. imported]; *p* < 1×10^-5^ for all), controlling for the level of local transmission. We found that the impact of interventions in the unions geographically closer or more connected to the western part of the CHT region (such as Lama) was higher than the unions with higher incidence but lower connectivity (such as Remakry) (Fig. 3). Our analysis therefore provides a quantitative measure of intervention effects for guiding targeted interventions and resource allocation in the CHT. As sensitivity analyses, we also repeated the analysis assuming 10%, 80% and 90% reporting rates for malaria incidence (Supplementary Fig. 4) and quantified the impact of interventions resulting in 50%, 80%, and 90% reductions of the mosquito to human ratio, as opposed to complete local elimination (Supplementary Fig. 5). Results with lower reporting rates were qualitatively similar to our main results. As expected, a smaller reduction in *m* leads to a lower percentage reduction in incidence due to intervention, but the results and patterns are qualitatively similar. Most notably, intervening in the same set of locations highlighted in Fig. 3B and C, have the largest impact in reducing overall incidence. For reporting rates of 80% and 90%, the top five locations were identical to those in the main analysis; under the 10% reporting rate, four locations overlapped, and the remaining one from the main top five (Chokhyong) ranked seventh.

## Discussion

Designing targeted malaria control interventions requires a careful understanding and quantification of how travel drives the spatial epidemiology of malaria, and the relative importance of local transmission and importation. Identifying source-sink dynamics and connectivity between locations can be helpful for control programs since the overall impact of controlling transmission in source locations depends on (1) the local transmission intensity and incidence, (2) the degree to which the location is connected to other locations; and (3) the population density and transmission intensity in the connected sink locations.

We constructed a metapopulation mechanistic model to estimate transmission intensity for each union and assess the potential impact of vector control. Since mobile phone data were not available in some of the forested areas, we utilized statistical models to infer missing mobility data, which enabled us to construct a metapopulation model with complete mobility network for the entire CHT region. We found that, in general, incidence in a particular location was associated with the effectiveness of intervention efforts in reducing overall malaria burden in the CHT. However, due to frequent travel between high and low transmission areas, vector control efforts in many areas with low incidence also had a moderate impact in reducing overall incidence, indicating that only focusing on high incidence areas is not sufficient for malaria control. We showed that regions with relatively high transmission intensity and higher connectivity with more populated regions in the west had a greater impact, and therefore prioritizing interventions in these regions are likely to be more effective.

Previous studies on spatial heterogeneity in malaria transmission have primarily focused on observed variations in incidence^1,18–22^, often without directly accounting for the impact of human mobility. However, our study, along with previous research^8,21^, demonstrates that human mobility can impact the interpretation of spatial heterogeneity in malaria transmission studies. Considering mobility when interpreting malaria transmission in Thailand suggested that the three main hotspots were not likely to be connected^21^. A study in Namibia revealed much greater levels of spatial heterogeneity of malaria transmission after incorporating human mobility^8^. The integration of parasite genetic data with human mobility data identified regions with a high proportion of domestically imported infections in the CHT region of Bangladesh^7^. The scarcity of studies incorporating human mobility may stem from the limited availability of mobility data, historically, particularly in forested areas where malaria transmission tends to be higher^23^. With mobility data becoming increasingly accessible in recent years^24^, our study provides a method to infer missing mobility data for regions with limited phone tower coverage, promoting the incorporation of human mobility in analyses of spatial heterogeneity in malaria transmission to enhance resource allocation. While our results for source-sink locations are specific to the CHT region, the methods we developed in this study are applicable to the entire country and beyond.

Our study has several important limitations. First, we used the steady-state solution to the metapopulation model, fit to observed incidence data, to estimate the local transmission intensity and proportion of imported infections. This requires the assumption of temporal stability in incidence. Until recently, malaria incidence has varied only moderately year to year in the CHT allowing us to approximate the steady-state; however, this model would not be appropriate for future planning efforts when large variations in incidence occur as has happened in 2021–2022^25^. Our modeling framework also ignores any possible seasonal effects, either in incidence, the vector population, or in human mobility—all of which have been shown to vary seasonally in prior research^1,26–29^. These seasonal variations could have interaction effects and the areas where vector control is most effective could change throughout the course of the year. Our model also ignores any spatial or temporal variations in malaria reporting rates, which could impact our results. For example, if malaria incidence were differently reported in forested regions versus urban region, we may be over or underestimating the impact of interventions depending on the discrepancy in reporting between these regions. Our results are also based on the travel behavior of mobile phone users and may not be reflective of the population in general. Although this is a well-established challenge of using mobile phone CDR data, previous research has suggested that biases due to demographic differences between mobile phone owners and the general population are likely to be small especially in places like Bangladesh with high mobile phone ownership^30^. We also used population sizes from the 2011 census^13^, which, though potentially outdated, is the only census data available. To infer travel for districts without mobile phone coverage we relied on a statistical model. However, the areas without CDR data were more remote and the travel patterns in those areas may not be accurately predicted by the fitted gravity model using CDR data from the more populated areas. Travel surveys, which were collected from malaria patients, can potentially be used to test the accuracy and generalizability of this method, and is a key direction for future work. Finally, there may also be discrepancies between a mobile phone subscriber’s actual location and the location assigned based on their nearest tower. Our observed incidence data may also be biased due to differential access to health care and testing across the CHT, as well as variations in the proportion of infections that are asymptomatic, which is likely higher in endemic high transmission areas. Moreover, while the proportion of *Plasmodium vivax* infections was historically low^31^, a recent study in Bandarban District, Bangladesh, found *P. vivax* infections throughout the study area, accounting for 42% (16/37) of mono-infections^32^. Since *P. vivax* can cause relapses, leading to bloodstream infections and potential transmission weeks, months, or even years after the initial mosquito bite, its increasing proportion poses additional challenges to malaria control efforts that were not considered in our study. Additionally, the lack of data on mosquito abundance and vectoral capacity made it difficult to validate the estimated mosquito-to-human ratio from our fitted mechanistic model. Finally, our results do not account for the cost of rolling out and scaling up malaria interventions, or potential non-linearities in the relationship between incidence reduction and the cost of interventions. Integrating our model results within a cost-effectiveness framework is an important direction for future research.

We provide the first quantitative measure for intervention effects in the CHT for policy makers to prioritize targeted interventions. Although we consider only one possible intervention scenario—vector control to eliminate the mosquito population—our fitted model could be applied to explore other intervention scenarios, such as simultaneous targeting of multiple locations, and be integrated with additional factors, such as the cost of interventions and the accessibility to diagnosis and treatment, to guide decision-making for future malaria control and elimination efforts.

## Supplementary information

Supplemental Material

Reporting Summary

## Data Availability

Access to mobility data is regulated through non-disclosure agreements (NDAs) and data sharing agreements, and cannot be released publicly. All requests for mobile phone dataset should be directed to Telenor Research, Norway. All model estimates and source data for recreating figures are available at https://zenodo.org/records/17060935.

## Code availability

All code used in this study is available at https://zenodo.org/records/17060935^33^.
