# Supplementary material for: Identifying malaria elimination strategies in the presence of human movement in Bangladesh
Source: Commun Med (Lond). 2025 Nov 7;5:461. doi: 10.1038/s43856-025-01145-6 (PMC12594956; doi:10.1038/s43856-025-01145-6)
Supplement: Supplementary file 1 — Supplemental Material [file 43856_2025_1145_MOESM1_ESM.pdf]

Identifying malaria elimination strategies in the presence of human movement in Bangladesh

Mahmud *et al.*

Supplementary materials

Parameter	Definition	Value	References
$\boldsymbol{m}$	Ratio of the total female mosquito population to the total human population	Estimated	
$\boldsymbol{a}$	biting rate of mosquitoes on humans	0.3	[8, 33, 34]
$\boldsymbol{b}$	Probability that an infectious mosquito bite transmits the disease to a human	0.1	[8, 33]
$\boldsymbol{c}$	Probability that a susceptible mosquito becomes infected after biting an infectious human	0.214	[8, 35, 36]
$\boldsymbol{r}$	Recovery rate of infected humans	1/150	[8, 37, 38]
$\boldsymbol{\mu}$	Mortality rate of infected mosquitoes	1/10	[8, 33]
$\boldsymbol{\tau}$	Incubation period within mosquitoes	10	[39]

Supplementary Table 1: Parameter descriptions and values for the metapopulation malaria transmission model.

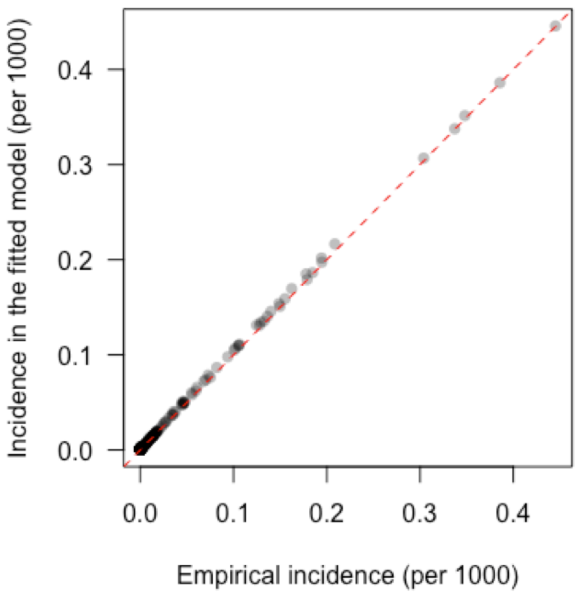

Supplementary Figure 1. Relationship betweeb empirical (observed) incidence data and the simulated incidence from the fitted model. Red dashed line shows the x=y line.

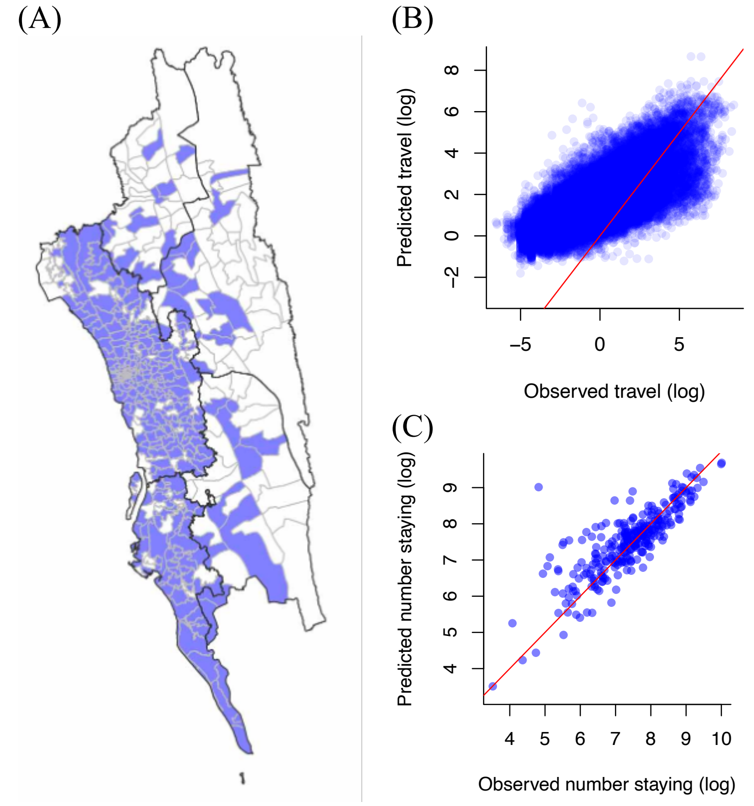

Supplementary Figure 2. (A) The geographic distribution of the union with (blue) or without (white) mobile phone calling data. Statistical fitting for the movement between unions (B) and the number of subscribers who remain in the same union (C). The observed mobility data in (B) and (C) come from the mobile phone call detail records (CDR), and the predicted values are derived from fitting Equations 1 and 2 to the CDR mobility data. The red lines in the plots represent the x=y line.

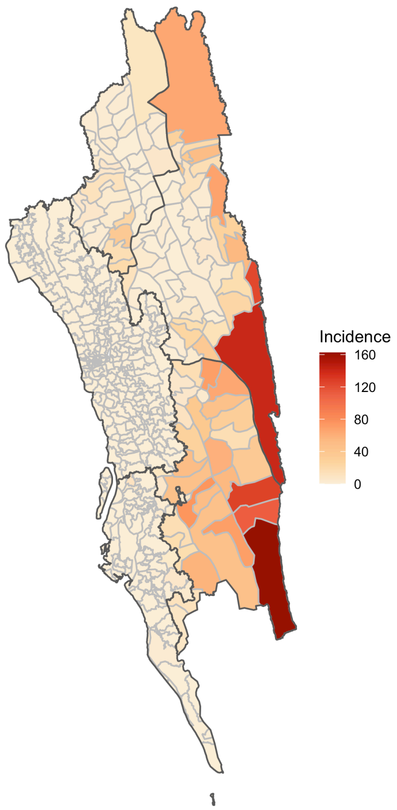

Supplementary Figure 3. Map of incidence (number of recorded cases per 1000 population per year) in the CHT.

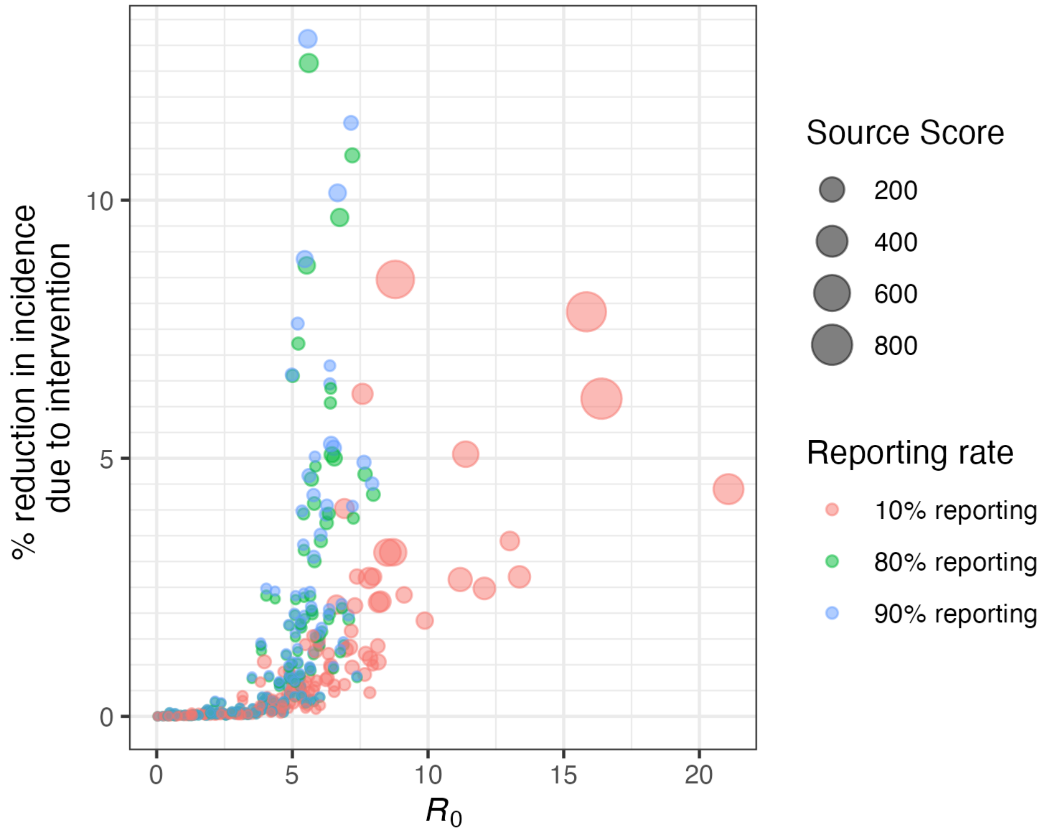

Supplementary Figure 4. The estimated local transmission intensity and source score for different levels of observed incidence reporting rates.

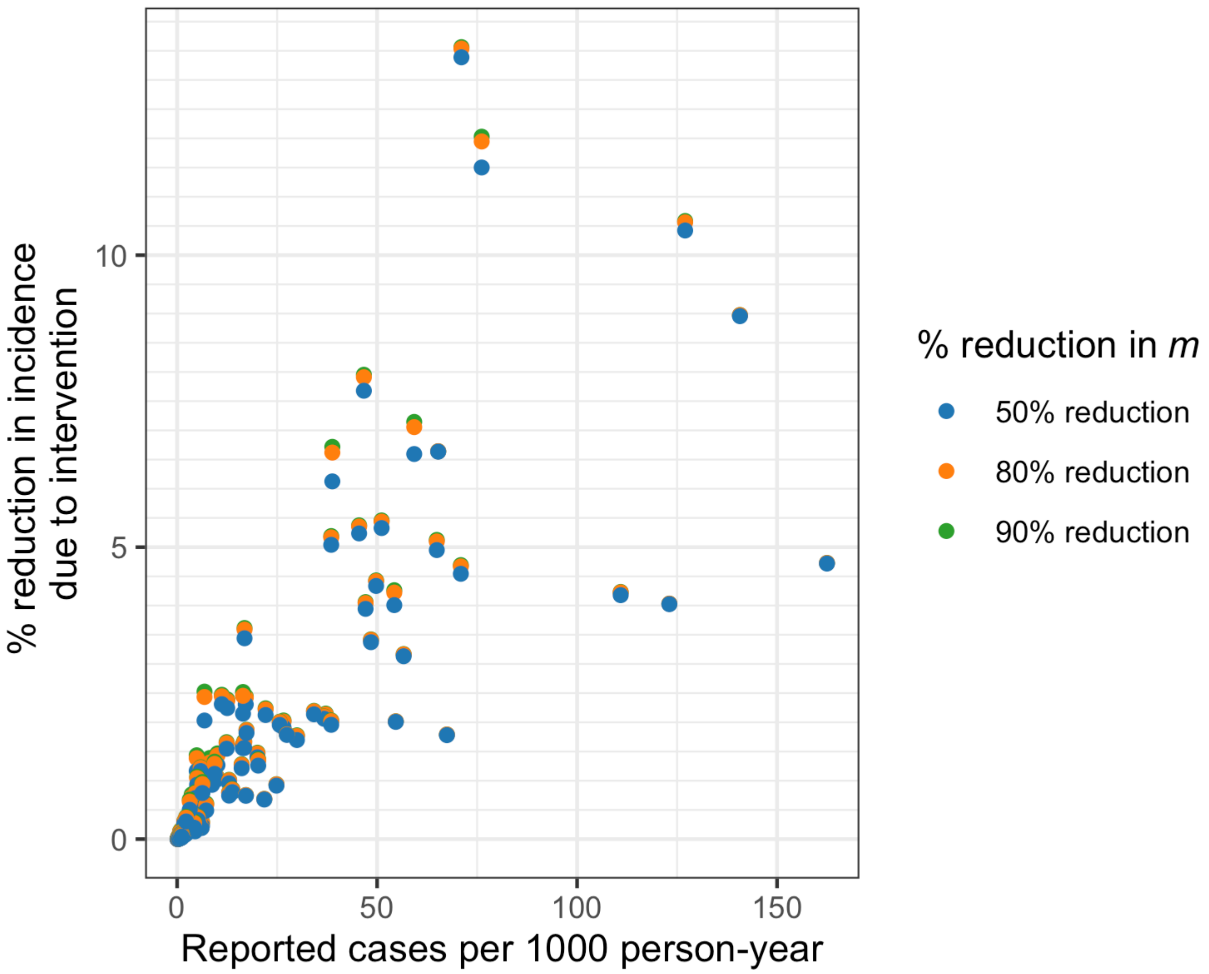

Supplementary Figure 5. The impact of the intervention by incidence (number of recorded cases per 1000 persons per year) of the location where the intervention is administered for different levels of reduction in the ratio of the total female mosquito population to the total human population, *m*.
